# General Anesthesia Using Laryngeal Mask Airway Ventilation With Low-Dose Neuromuscular Blockade Is Safe and Effective for Patients Undergoing Hip Arthroscopy

**DOI:** 10.1016/j.asmr.2025.101218

**Published:** 2025-07-08

**Authors:** Justin T. Jabara, Jillian H. Neuner, Akash Vasanthan, Pedram Aleshi, Sakura Kinjo, Stephanie E. Wong, Alan L. Zhang

**Affiliations:** a Department of Orthopaedic Surgery, University of California-San Francisco, San Francisco, California, U.S.A.; b School of Medicine, University of California-San Francisco, San Francisco, California, U.S.A.; c Department of Anesthesiology, University of California-San Francisco, San Francisco, California, U.S.A.

## Abstract

**Purpose:**

To investigate safety and efficacy of a general anesthesia protocol using laryngeal mask airway (LMA) ventilation without endotracheal intubation (ETT) in the setting of a low dosage of neuromuscular blockade (NMBA) for patients undergoing hip arthroscopy.

**Methods:**

Patients who underwent hip arthroscopy for femoroacetabular impingement syndrome at a single institution from 2014 to 2024 were retrospectively analyzed. Those who underwent hip arthroscopy for non-femoroacetabular impingement syndrome indications and those with a concomitant periacetabular osteotomy were excluded. General anesthesia was administered with propofol and sevoflurane, with LMA ventilation. A low-dose (10-50 mg) NMBA agent (rocuronium) was given before hip distraction. The primary outcome of interest was pulmonary aspiration. Secondary outcomes of interest included conversion to ETT and operating room time.

**Results:**

This study included 1,169 cases (49.8% female) with mean age 37.8 years (± 11.0) and body mass index 24.4 (± 3.9). In total, 64.0% were American Society of Anesthesiologists (ASA) class I, 34.8% class II, and 1.2% class III. In 24 cases, patients underwent ETT without attempting LMA as a result of preoperative aspiration risk factors. Of the 1,145 cases that underwent LMA with NMBA, mean dose of rocuronium given was 20.4 mg, and no patients experienced pulmonary aspiration or anesthesia complications. Twenty-one cases (1.8%) required conversion to ETT as the result of poor LMA fit or ventilation leakage. ASA class was not associated with LMA-to-ETT conversion (*P* = .77). Mean airway manipulation time was 6.0 minutes (± 6.4), mean procedure duration was 100.3 minutes (± 48.7), and mean procedure end to airway removal time- 5.0 minutes (± 5.0). No surgical complications related to hip distraction or traction force occurred.

**Conclusions:**

Administration of general anesthesia using LMA ventilation with a low dosage of neuromuscular blockade is safe and effective for patients undergoing hip arthroscopy. The rate of conversion to endotracheal intubation was low and showed no association with ASA classification.

**Level of Evidence:**

Level IV, retrospective therapeutic case series.

Hip arthroscopy increasingly is performed in outpatient settings in the United States.^1^ Protocols to improve patient safety and surgical efficiency are evolving. The use of a neuromuscular blocking agent (NMBA) can help facilitate the distraction of the hip joint.^2^ These agents decrease the force required for adequate distraction of the hip joint, which improves patient safety, because increased duration and applied traction force are associated with nerve palsy.^3^^,^^4^

Variability in anesthesia practice exists regarding airway management with the use of NMBAs (i.e., rocuronium), with many providers preferring endotracheal intubation over laryngeal mask airway (LMA) ventilation. Compared with LMA, the use of endotracheal tubes (ETTs) can result in an increased incidence of dysphonia^5^ and pharyngeal discomfort.^6^ LMA can be easier and faster to insert in some patients,^7^^,^^8^ with lower postairway removal morbidity reported.^9^ However, the primary concern with using rocuronium without an ETT is the risk of gastric insufflation during positive pressure ventilation. This ventilation technique is often necessary when NMBAs are used, because they induce diaphragmatic paralysis. Positive pressure ventilation with an LMA may increase the chance of insufflation of the stomach, resulting in a theoretical increased risk of pulmonary aspiration.^10^, ^11^, ^12^, ^13^, ^14^ Other risk factors for aspiration include body mass index (BMI), previous gastric/esophageal surgery, gastrointestinal obstruction, delayed gastric emptying, and glucagon-like peptide-1 receptor agonists such as semaglutide.^15^, ^16^, ^17^ Because the use of LMA ventilation during hip arthroscopy may improve operating room efficiency and decrease the side effects associated with endotracheal intubation, it may be valuable to evaluate the risk of aspiration when an LMA is used in conjunction with neuromuscular blockage.^6^^,^^8^^,^^18^^,^^19^

The purpose of this study was to investigate the safety and efficacy of a general anesthesia protocol using LMA ventilation without endotracheal intubation in the setting of a low dosage (10-50 mg) of neuromuscular blockage for patients undergoing hip arthroscopy surgery. We hypothesized that no major adverse complications, pulmonary aspiration being the primary outcome of interest, would be observed in patients undergoing hip arthroscopy with a combined NMBA-LMA anesthetic protocol.

## Methods

This study was approved by the institutional review board (24-42503).

### Patient Selection

Patients were included if they underwent hip arthroscopy for femoroacetabular impingement syndrome (FAIS) between January 2014 and September 2024. Exclusion criteria included hip arthroscopy for non-FAIS indications and patients who underwent concomitant periacetabular osteotomy. This study was approved by the institutional review board.

### Anesthesia Protocol

Patients undergoing hip arthroscopy were seen in a preoperative anesthesia clinic. Risk factors for pulmonary aspiration (i.e., obesity and obstructive sleep apnea) and other anesthetic considerations were evaluated at this time. Patients taking glucagon-like peptide 1 receptor agonists (semaglutide) were instructed to pause taking their medication 10 days before surgery. Intravenous induction of general anesthesia was performed using propofol, with time and dosage depending on anesthesiologists’ preference. An LMA was placed postinduction, before the administration of NMBAs. LMA types available were as follows: air-Q (CookGas; Mercury Medical, Clearwater, FL), i-gel (Intersurgical, Wokingham, England), LMA-Classic (Teleflex, Morrisville, NC), LMA-Supreme (Teleflex), LMA-Unique (Teleflex), and LMA-ProSeal (Teleflex). After the LMA was secured with tape, the patient was positioned into traction boots. Once positioned, a series of fluoroscopic images were taken. After completion of imaging, the anesthesia provider administered rocuronium, typically 10 to 50 mg. Rocuronium dosage was based on anesthesiologist preference: either weight based (0.5 mg/kg) or starting with an initial 20-mg dose and then redosed in 10-mg increments up to 50 mg if central compartment distraction was difficult or muscle spasm occurred. The most commonly used ventilator mode was pressure support ventilation, and transitioned to a controlled mechanical ventilation mode, most often synchronized intermittent mandatory ventilation-pressure control volume guarantee mode, if diaphragmatic paralysis occurred after rocuronium administration. At the conclusion of the procedure, sugammadex was administered in a weight-based fashion (typically 2 mg/kg; however, a practical dose of 200 mg used frequently because of vial size) if indicated for reversal of rocuronium.

### Surgical Technique

All patients included in the study were indicated for hip arthroscopy of the central compartment for FAIS. Surgeries were performed in an ambulatory surgery center by 2 fellowship-trained orthopaedic sports surgeons with expertise in hip arthroscopy (A.L.Z. and S.E.W.). Patients were positioned supine on a postless table specific for hip arthroscopy (Pivot Guardian; Stryker, Kalamazoo, MI) or a hip distraction attachment with a large diameter perineal post (Hip Positioning System; Smith & Nephew, Andover, MA). After induction of general anesthesia and securing of the airway, a 17-gauge spinal needle was used to perform an air arthrogram to aid in distraction of the hip joint.^20^ After fluoroscopic confirmation of the air arthrogram, manual traction was applied across the central compartment. After fluoroscopic confirmation of hip distraction, fluoroscopy was used to gain central compartment access first through an anterolateral portal, and then a midanterior portal. Interportal or periportal capsulotomy was performed according to surgeon preference and patient selection. Diagnostic arthroscopy was performed, followed by arthroscopic labral repair, debridement, or reconstruction. Acetabuloplasty was performed when indicated. After central compartment pathology was addressed, traction was released, and peripheral compartment pathology was managed (femoroplasty). Closure of the hip capsule was performed if interportal capsulotomy was elected or on a case-by-case basis when periportal capsulotomy was used.

Patients were assessed for age, weight, BMI, history of gastric/esophageal surgery, hiatal hernia, American Society of Anesthesiologists (ASA) physical status classification, and symptomatic gastroesophageal reflux disease. The primary outcome investigated in this study was pulmonary aspiration, with secondary outcomes being conversion to endotracheal intubation and surgical times.

### Statistical Analysis

χ^2^ analysis was employed to evaluate associations between categorical variables, with a significance level set at *P* < .05.

## Results

### Demographic and Anesthesia Characteristics

The sample included 1,169 cases of hip arthroscopy, of which 1,014 unique patients were identified. This series of hips included 582 female (49.8%) and 587 male (50.2%) patients. The mean age of the study population was 37.8 years (± 11.0), and the mean BMI was 24.4 (± 3.9). No patients had a history of hiatal hernia or previous gastric/esophageal surgery.

ASA categories were as follows for the study population at the time of surgery: class I (normal, healthy patient) 748 (64.0%), class II (patient with mild systemic disease) 407 (34.8%), class III (patient with severe systemic disease) 14 (1.2%) (Table 1). Of cases where the LMA design was recorded in the medical record (n = 948), the most common LMA types were as follows: i-gel (484, 51.1%), LMA-Unique (172, 18.1%), LMA-Supreme (132, 13.9%), LMA-ProSeal (108, 11.4%), LMA-Classic (51, 5.4%), and Air-Q (1, 0.1%).

**Table 1:** Preoperative Demographic Characteristics (n = 1,169)

Characteristic	Results
Sex	
Female	582 (49.8)
Male	587 (50.2)
Age, yr	37.8 ± 11.0
ASA status	
Class I	748 (64.0)
Class II	407 (34.8)
Class III	14 (1.2)
BMI	24.4 ± 3.9
History of gastric or esophageal surgery	0
Presence of hiatal hernia	0

NOTE. Continuous data listed as mean ± standard deviation. Nominal data listed as number (%).

ASA, American Society of Anesthesiologists; BMI, body mass index.

**Fig 1:**
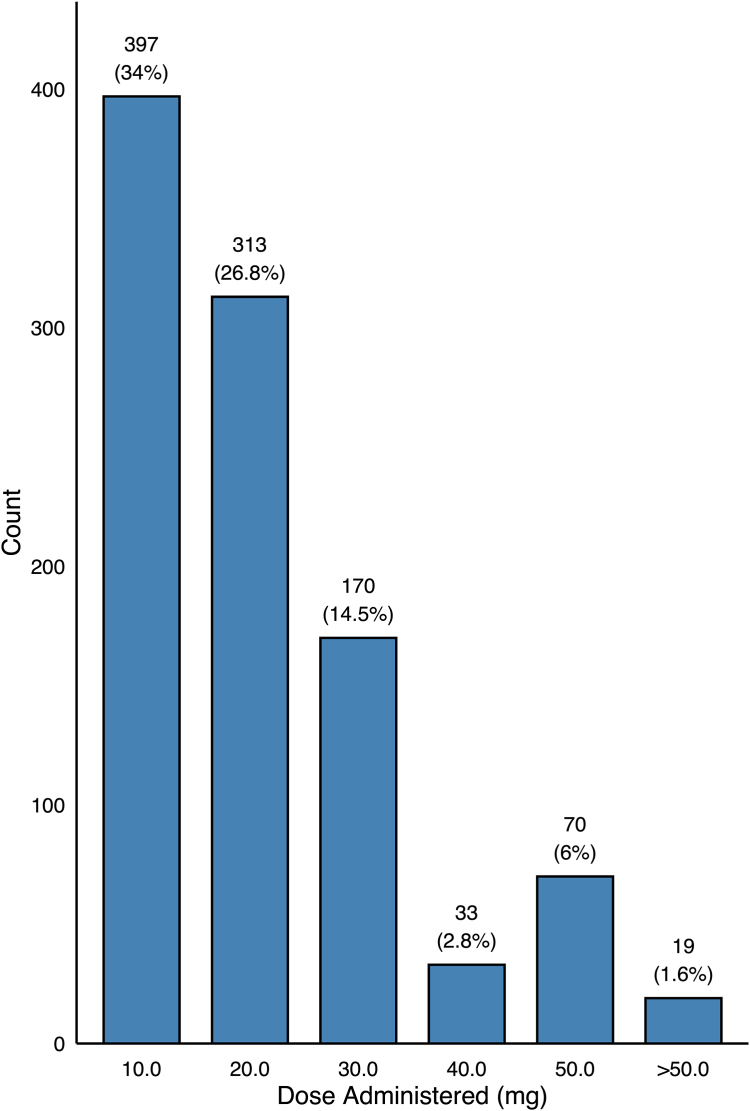
Distribution of common dosages of rocuronium (neuromuscular blocking agent) administered to patients during hip arthroscopy.

Every patient received a dose of rocuronium. 397 (34.0%) received a dose of 10 mg, 78 (6.7%) received 15 mg, 313 (26.8%) received 20 mg, 32 (2.7%) received 25 mg, 170 (14.5%) received 30 mg, 3 (0.3%) received 35 mg, 33 (2.8%) received 40 mg, and 70 (6.0%) received 50 mg. 54 (4.6%) received a dose less than 10 mg, and 19 (1.6%) received a dose more than 50 mg; the minimum dose was 2.0 mg, and the maximum dose was 100 mg. The mean dosage was 20.4 mg (± 13.2) (Fig 1). In total, 536 (45.9%) patients received sugammadex at the conclusion of case. 61.2% received 100 or 200 mg, with a mean dose of 168.5 mg (± 52.7).

In 24 of 1,169 cases (2.1%), an ETT was placed without an LMA attempt. In this cohort of patients, the following comorbidities were reported on their preoperative anesthesia evaluation: 10 of 24 symptomatic gastroesophageal reflux disease, 9 of 24 snoring, 4 of 24 obstructive sleep apnea, 14 of 24 obesity (BMI ≥30), 6 of 24 thyromental distance between 4.5 and 6 cm, 1 of 24 history of unilateral Bell palsy, and 3 of 24 Mallampati class III. In total 16 of 24 patients were documented as having at least 2 of these reported risk factors (Table 2).

**Table 2:** Preoperative Anesthesia Characteristics of Patients Who Underwent Endotracheal Intubation (n = 24)

Characteristic	Results
Symptomatic GERD	10 (41.7)
History of snoring	9 (37.5)
Obstructive sleep apnea	4 (16.7)
Obesity (BMI ≥30)	14 (58.3)
Thyromental distance 4.5-6 cm	6 (25.0)
History of unilateral Bell palsy	1 (4.2)
Mallampati class III	3 (12.5)
Two or more aspiration risk factors	16 (66.7)

NOTE. Nominal data listed as number (%).

BMI, body mass index; GERD, gastroesophageal reflux disease.

### Operative Times

The mean time spent manipulating the airway was 6.0 minutes (± 6.4). The procedure duration was a mean of 100.3 minutes (± 48.7), and the mean procedure end time to airway removal time was 5.0 minutes (± 5.0) (Table 3).

**Table 3:** Operative Times

Characteristic	Minutes
Manipulating airway	6.0 ± 6.4
Procedure duration	100.3 ± 48.7
Procedure end to airway removal	5.0 ± 5.0

NOTE. Data listed as mean ± standard deviation.

### Intraoperative and Postoperative Complications

In 21 of 1,145 cases (1.8%), intraoperative conversion to endotracheal intubation was required. A reason for conversion was documented in 7 of 21 patients. The reason for conversion to ETT was reported in 6 of these patients as a leaking LMA. One anesthesia provider was not comfortable administering rocuronium with an LMA in place. There was no association between ASA class and conversion from LMA to ETT (*P* = .77). There was no difference in LMA to ETT conversion between male (2.4%) and female (1.2%) patients (*P* = .193). No patients experienced pulmonary aspiration or anesthesia complications, including dental or vocal cord injuries. There were no surgical complications due to inadequate hip distraction nor difficultly with joint access due to lack of muscle paralysis.

## Discussion

The most important finding of this study was that we found no major complications from using low-dose neuromuscular blockade agents with LMA during outpatient hip arthroscopy. No pulmonary aspiration was observed, and the rate of conversion from LMA to endotracheal intubation was low, at 1.8%.

There has been longstanding debate regarding the safety profile of neuromuscular blockade agent usage without an endotracheal tube.^11^^,^^21^^,^^22^ Although previous studies have demonstrated the safety profile of LMA used with neuromuscular blockade agents,^6^^,^^10^^,^^13^ in this study we specifically demonstrate the safety profile of hip arthroscopy combined with the described anesthetic technique. The NMBA-LMA protocol used by our institution is safe and effective, evident by the 0% aspiration rate. This compares to Bernardini and Natalini^10^ reporting an aspiration rate of 0.02% with LMA using positive pressure ventilation. This outcome is partly attributed to the standardized process of screening patients preoperatively for aspiration risk factors. Known patient aspiration risk factors include BMI, previous gastric/esophageal surgery, gastrointestinal obstruction, delayed gastric emptying, and use of glucagon-like peptide-1 receptor agonists.^23^ The study population consisted of mainly young, healthy patients without upper gastrointestinal comorbidity. Patients were instructed to hold glucagon-like peptide-1 receptor agonists for 10 days before surgery. Twenty-four patients in the present study underwent endotracheal tube placement without an attempt at an LMA, as decided by the anesthesia provider. Of these patients 16 of 24 had at least 2 reported risk factors for aspiration. The results of this study should not be generalized to patients with a substantially elevated BMI or multiple other risk factors. Epidemiologic studies have demonstrated the average BMI in a patient undergoing hip arthroscopy to be 25.1 (range 15-53), and the mean age at time of arthroscopy to be 28.^24^ The authors believe conclusions can be applied to a typical young and healthy hip arthroscopy patient population if patients are appropriately screened at the time of preoperative anesthesia evaluation.

LMA-to-ETT conversion occurred in 1.8% of cases (21/1145), with no ASA class association. The reported reason for conversion was mainly persistent leak and difficult LMA seating. The most commonly used LMA device was the i-gel (Intersurgical). The i-gel contains a solid, noninflatable cuff made of a thermoplastic elastomer with a gel-like texture at the tip, a short airway tube, and a gastric drainage channel.^25^^,^^26^ The absence of an inflatable cuff may allow for more efficient seating in patients. Having multiple modern LMA devices available to ensure an appropriate seal may be an important factor in keeping the rate of conversion to endotracheal intubation low. Advancements in LMA designs have focused on structural modifications, such as improved cuff shapes, enhanced material compliance, and the incorporation of gastric drainage channels to enhance performance, particularly in patients with diminished muscle tone due to muscle relaxation.^27^^,^^28^ Some designs integrate the inflation tube into the airway shaft, reducing the risk of kinking or occlusion.^29^ Additional modifications have replaced inflatable cuffs with gel-filled cuffs that mold to the airway, offering an effective seal without the need for cuff inflation.^25^^,^^30^ Furthermore, LMAs now include gastric drainage channels, which allow for passive venting of gastric contents, reducing the risk of aspiration during positive pressure ventilation.^31^^,^^32^ Successful LMA use depends on correct size and cuff selection for optimal ventilation, and having a wide selection is important for proper seating.^33^ In addition, some LMA devices may show a decreased risk of pulmonary aspiration.^34^

Compared with ETT, the LMA has been shown to be easier and faster to insert in some studies^7^ and has a lower rate of dysphonia,^5^ pharyngeal discomfort,^6^ and laryngospasm^9^ at airway removal. Although meta-analysis shows no time differences between airways,^9^ some authors report longer induction times (7.4 vs 5.8 minutes)^8^ and longer extubation times (12 vs 7.2 minutes) with ETT.^35^ Our data show a mean induction time of 6.0 minutes and LMA removal time of 5.0 minutes, supporting an efficient NMBA-LMA workflow for outpatient surgery. Given the increased risks airway morbidity associated with endotracheal intubation as compared to LMA,^9^^,^^36^ the authors of this study believe this anesthetic technique offers a valuable resource for improving patient satisfaction while creating an efficient outpatient hip arthroscopy protocol.

The findings of the present study are important because neuromuscular blockade agents facilitate atraumatic distraction of the hip joint, allowing access to the central compartment.^2^ The relatively low mean dose of rocuronium administered (20.4 mg) was sufficient for hip distraction, and there were no distraction difficulties nor traction injuries noted. This dose contrasts with endotracheal intubation using rocuronium, which typically requires a greater dose of 0.6 to 1.2 mg/kg, with a common induction dose of 50 to 100 mg for adults. The greater dose ensures sufficient neuromuscular blockade to facilitate direct laryngoscopy and secure an endotracheal tube. In contrast, LMA placement can often be performed without the use of a paralytic, as the device is designed to sit supraglottically rather than pass through the vocal cords, reducing the need for deep muscle relaxation. This environment allows for successful hip distraction with only a low dose administration of rocuronium at the time traction is applied. This substantial contrast in dosing highlights the more invasive nature of endotracheal intubation with greater complication rates. Lower dosages used with this protocol potentially reduce recovery time and the risk of prolonged neuromuscular blockade. In our series, Sugammadex was administered in 536 cases (45.9%) when persistent neuromuscular blockade was present at the end of the procedure.

### Limitations

This study is not without limitations. This is a single-institution protocol at a high-volume hip arthroscopy center, and results may not be generalizable to other surgery centers without expertise in hip arthroscopy. Further, no control group is present, and this case series is not sufficient to claim superiority of LMA compared with ETT for airway management during hip arthroscopy. Selection bias may be present, as the anesthesiologist might have preoperatively elected to use an ETT instead of an LMA due to inherent patient airway risk factors, potentially influencing outcomes. It should also be noted that some patients did not have a documented reason if treated with endotracheal intubation. Additional limitations include variability in the induction anesthetic protocol as well as variability in types of LMA masks that were used. In addition, this series was performed at an institution where airway management with LMA is routinely used, contributing to anesthesiologist familiarity and expertise with this protocol. Future studies should evaluate outcomes at centers currently using NMBA-ETT for hip arthroscopy and whether improvements are seen with conversion to NMBA-LMA.

## Conclusions

Administration of general anesthesia using LMA ventilation with a low dosage of neuromuscular blockade is safe and effective for patients undergoing hip arthroscopy. The rate of conversion to endotracheal intubation was low and showed no association with ASA classification.

## Disclosures

The authors declare the following financial interests/personal relationships which may be considered as potential competing interests: A.L.Z. reports board or committee member of American Academy of Orthopaedic Surgeons, American Orthopaedic Society for Sports Medicine, and Arthroscopy Association of North America; editorial or governing board of *Arthroscop*y and *The American Journal of Sports Medicine*; other financial or material support from CONMED Linvatec; and paid consultant for DePuy, A 10.13039/100004331Johnson & Johnson Company and Stryker. SEW reports Editor in Chief of *Current Reviews in Musculoskeletal Medicine*. All other authors (J.T.J., J.H.N., A.V., P.A., S.K.) declare that they have no known competing financial interests or personal relationships that could have appeared to influence the work reported in this paper.
